# iPTT(2 L)-CNN: A Two-Layer Predictor for Identifying Promoters and Their Types in Plant Genomes by Convolutional Neural Network

**DOI:** 10.1155/2021/6636350

**Published:** 2021-01-05

**Authors:** Ang Sun, Xuan Xiao, Zhaochun Xu

**Affiliations:** Jing-De-Zhen Ceramic Institute, Jingdezhen, China

## Abstract

A promoter is a short DNA sequence near to the start codon, responsible for initiating transcription of a specific gene in genome. The accurate recognition of promoters has great significance for a better understanding of the transcriptional regulation. Because of their importance in the process of biological transcriptional regulation, there is an urgent need to develop in silico tools to identify promoters and their types timely and accurately. A number of prediction methods had been developed in this regard; however, almost all of them were merely used for identifying promoters and their strength or sigma types. Owing to that TATA box region in TATA promoter that influences posttranscriptional processes, in the current study, we developed a two-layer predictor called iPTT(2L)-CNN by using the convolutional neural network (CNN) for identifying TATA and TATA-less promoters. The first layer can be used to identify a given DNA sequence as a promoter or nonpromoter. The second layer is used to identify whether the recognized promoter is TATA promoter or not. The 5-fold crossvalidation and independent testing results demonstrate that the constructed predictor is promising for identifying promoter and classifying TATA and TATA-less promoter. Furthermore, to make it easier for most experimental scientists get the results they need, a user-friendly web server has been established at http://www.jci-bioinfo.cn/iPPT(2L)-CNN.

## 1. Introduction

Promoters are usually short sequences containing the transcription start site (TSS) and some regulatory elements, which can determine under what conditions and where the transcription of a particular gene in genome is initiated [1]. During this process, the TATA box as one of *cis*-acting promoter sequences plays an important role in specifying transcription initiation sites and in promoter activation [2]. The TATA box is a short A+T-rich DNA sequence, which is conserved among most genes of eukaryotes and archaea. Histones and transcription factor proteins can bind to the TATA box region in the core promoter, thereby playing an important role in preventing and promoting the initiation of transcription, respectively. In the initiation of transcription in vitro, the first step is that the transcription initiation factor binds to the TATA box [3, 4], whereas deletion of the TATA box could result in zero or significantly reduced transcription levels [5]. The absence of a TATA box could generate transcripts at low levels with heterogeneous 5′ ends as characteristic [6]. Therefore, accurate identification of promoters in plant genomes and classifying their types, especially for TATA and TATA-less promoters, has great significance for a better understanding of the regulation of the plant gene expression [7].

The advances in high-throughput whole-genome sequencing and the accumulation of promoter sequences conformed by experiments have led to the emergence of databases, such as RegulonDB [8], PlantProm [9], and DBTSS [10], which could provide valuable information for training computational predictors for identifying and classifying promoters.

Actually, over the past few years, a number of such computational predictors had been developed [11–14]. To identify the sigma 54 promoters, a predictor named “iPro54-PseKNC” [15] was proposed by using pseudo k-tuple nucleotide composition (PseKNC). Recently, to identify promoters and their six types labeled by different sigma factors, many efforts had been made. Liu et al. [16] first constructed iPromoter-2 L based on multiwindow-based PseKNC and obtained stable prediction performance. Subsequently, for achieving a better prediction performance, Zhang et al. [17] fused multifeatures and adopted the F-score feature selection method to present a multilayer computational approach dubbed MULTiPly. Liu and Li [18] combined the smoothing cutting window algorithm and sequence-based features to improve the prediction performance again. Amin et al. [19] constructed a CNN-based classifier named iPromoter-BnCNN by combining sequence-order information and structural properties. Lyu et al. [20] presented a two-layer predictor called iPro2L-PSTKNC using position specific of nucleotide composition, currently achieving the best prediction performance. In addition, Xiao et al. [21] constructed a two-layer predictor iPSW(2 L)-PseKNC for identifying promoters and their strength. Umarov et al. [22] developed a predictor PromID to predict the exact location of the TSS within the genomic sequences detecting every possible position. Mishra et al. [23] presented a novel model called SEProm for predicting prokaryotic promoter based on the DNA structure and energetics.

Remarkable development have achieved in the important field of promoter identification and their type classification. However, all most methods have focused on classification sigma promoters, classification of TATA, and TATA-less promoters that are seldom touched. Zou et al. [1] proposed the SVM-based model by integrating multifeatures including GC skew, local word content, and DNA geometric flexibility to predict the two types of promoters. Furthermore, Ramzan et al. [24] developed a CNN model named CNNprom to recognize TATA and TATA-less promoters of Arabidopsis. However, exiting predictors have following shortcomings. (i) Not all the studies established user-friendly and publicly accessible web server, such as the SVM-based model proposed by Zou et al. [1], thereby causing much inconvenience to practical use for most experimental scientists. (ii) The datasets for constructing the training model only consist of promoter sequences of Arabidopsis, however, without promoter sequences of other plants such as Zea mays, an essential staple cereal crop. (iii) No crossspecies analysis is implemented in the abovementioned studies.

In the current study, we devoted to overcome the aforementioned disadvantages for improving the prediction capability in identifying TATA and TATA-less promoters of Zea mays genomes. At first, high-quality benchmark datasets confirmed by experiment were constructed. Subsequently, we analyzed sequence characteristics of plant promoters using the convolutional neural network (CNN) and developed a two-layer predictor called “iPPT(2 L)-CNN.” Its first layer can be used to identify whether a given query DNA sequence is of promoter or not, while its second layer is used to identify whether the recognized promoter belong to TATA or TATA-less promoter. And then, the crossvalidation test was used to evaluate our method. Finally, in according to the constructed model, a web server dubbed iPPT(2 L)-CNN was established.

## 2. Materials and Methods

### 2.1. Benchmark Dataset

**Figure 1:**
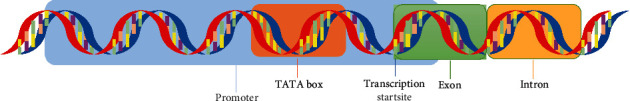
A schematic diagram of the locations of the promoter.

To construct a high-quality benchmark dataset, we downloaded TATA and TATA-less promoter sequences of Zea mays from the database EPDnew [25], which consists of eukaryotic promoters validated by experiments. A promoter region of a given size around the known TSS (from -200 bp to +50 bp, where +1 is a TSS position) is considered to be a positive sequence. A schematic diagram of the locations of the promoter is shown in Figure 1. The sequence segments with 251 bp were randomly selected from the nonpromoter sequence part as negative sequences. The ratio of the formed positive dataset and negative dataset is about 2 : 1 ratio. Moreover, we used the CD-HIT software [26] with the threshold value at 0.8 to remove redundancy, thereby reducing homologous bias [27]. Thus, the final benchmark dataset *S* could be obtained, as expressed by the following formulation.

(1) $$\begin{array}{c} S=S^{+}+S^{-}, \end{array}$$

where *S*^+^ represents the positive set containing 8,935 promoter sequences, while *S*^−^ represents the negative set composed of 17,606 nonpromoter samples. The symbol ∪ represents the union in the set theory.

In Eq. (1), the positive set *S*^+^ can be formulated by

(2) $$\begin{array}{c} S^{+}=S_{\text{TATA}}^{+}+S_{\text{TATA}-\text{less}}^{+}, \end{array}$$

where *S*_TATA_^+^ represents the positive subset containing 1,559 TATA promoters, while *S*_TATA_less_^+^ denotes the positive subset composed of 7,376 TATA-less promoters.

For constructing and training the prediction model, we randomly selected 80% the benchmark data as training dataset and the remained 20% as testing dataset to evaluate the proposed model. The benchmark dataset thus obtained can be downloaded from the web http://www.jci-bioinfo.cn/iPTT(2L)-CNN/download.

### 2.2. Nucleotide Representation

Each nucleotide in a sample sequence is represented by a 4-dimensional one-hot vector, which is a vector of zeros with a single one [28]. For example, nucleotide A is encoded by (1, 0, 0, 0); C (0, 1, 0, 0); G (0, 0, 1, 0); and T (0, 0, 0, 1). Thus, each sample sequence could be represented by a (4, 251) two-dimensional vector.

### 2.3. Two-Layer Classification Framework

**Figure 2:**
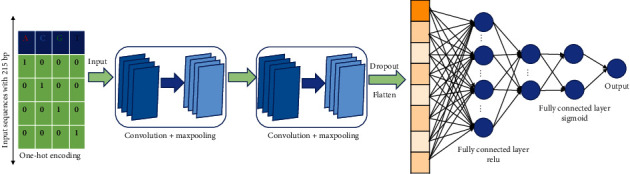
Schematic overview of the CNN model.

To make the prediction method not only available for identifying whether a DNA sample is of promoter or not but also able to identify its type, we developed a two-layer predictor. In fact, the two-layer classification framework has achieved remarkable successes in identification of membrane proteins and their types [29] as well as identification of the enhancers and their strength [30]. Furthermore, recent developments in deep learning, especially for CNN, have created fertile ground for the development of bioinformatics, particular for sequence analysis [31–38] and biological images [39]. Motivated by these successes, we used CNN as the classification framework to identify promoter and their types. The flow chart of the CNN model is shown in Figure 2.

Our architecture consists of two convolutional layers which are in series. The first convolutional layer consists of 300 filters with a filter size of 4. After the first convolutional layer, a max-pooling layer is followed. The output from the max-pooling layer is fed into the second convolutional layer consists of 120 filters with a filter size of 4. After the second convolutional layer, a max-pooling layer is followed. The output from the second max-pooling layer is concatenated, flattened, and fed into two standard fully connected layers, which contains 1,280,256 neurons in turn with the ReLU activation function. The outputs of the fully connected layers are fed into an output layer with the sigmoid activation function that provide the predictive likelihood of an input sequence.

Weight decay and dropout are used to improve the generalization capability of our model. Weight decay could effectively limit the number of free parameters in the model to avoid overfitting [22]. Furthermore, the variables in the two fully connected layers are randomly turned off during training process with probabilities of 0.5. These parameters were optimized by a standard 5-fold crossvalidation based on the MCC in the dataset. The specific parameters in our model are shown in Table 1.

**Figure 3:**
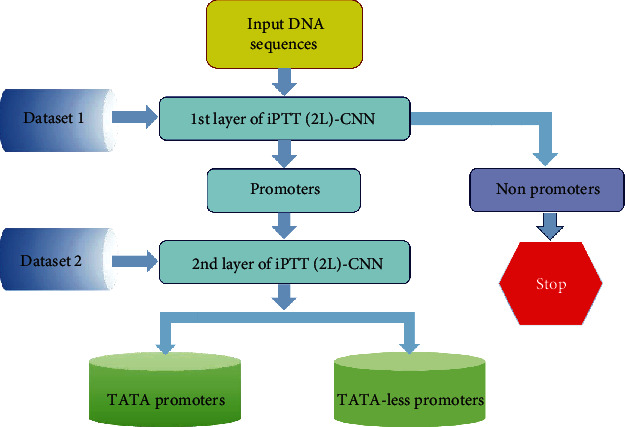
Overall workflow of iPTT(2 L)-CNN. Dataset 1 represents the dataset formulated by Eq. (1), and Dataset 2 means the dataset expressed by Eq. (2).

The predictor thus obtained is called iPTT(2 L)-CNN, where “i” stands for “identify”, “P” for “promoters”, “T” for “type”, “T” for “TATA”, and “2 L” for “two-layer”. The 1st layer serves to predict whether a query DNA sequence sample is of promoter or not, while the 2nd layer to further identify whether the recognized promoter is TATA and TATA-less promoter or not. A flowchart to show how the two-layer classifier works is given in Figure 3.

### 2.4. Performance Evaluation

The *K*-fold crossvalidation method is widely used in evaluating the anticipated accuracy of the predictor [28, 40–42]. In this study, 5-fold cross-validation was adopted to evaluate prediction quality. The performance of the proposed model for identifying the promoters and their types can be defined by the following common four metrics:

(3) $$\begin{array}{c} \left\{\begin{array}{c} S_{n}=1-\frac{N_{-}^{+}}{N^{+}},0\leq S_{n}\leq 1\\ S_{p}=1-\frac{N_{+}^{-}}{N^{-}},0\leq S_{p}\leq 1\\ \text{Acc}=1-\frac{N_{-}^{+}+N_{+}^{-}}{N^{+}+N^{-}},0\leq \text{Acc}\leq 1\\ \text{MCC}=\frac{1-\left(\left(N_{-}^{+}/N^{+}\right)+\left(N_{+}^{-}/N^{-}\right)\right)}{\sqrt{\left(1+\left(N_{+}^{-}-N_{-}^{+}/N^{+}\right)\right)\left(1+\left(N_{-}^{+}-N_{+}^{-}/N^{-}\right)\right)}},-1\leq \text{Mcc}\leq 1 \end{array}\right., \end{array}$$

where *N*^+^ is the total number of the positive samples, while *N*^−^ represents the total number of the negative samples; *N*_+_^−^ is the number of the negative samples incorrectly predicted to be of positive samples, while *N*_−_^+^ is the number of positive samples incorrectly predicted to be of negative samples.

In addition, AUROC is a popular metric for evaluate performance of the proposed models. According to the area under 1-specificity and sensitivity curves, AUROC values could be calculated.

## 3. Results and Discussion

### 3.1. Parameter Settings

**Figure 4:**
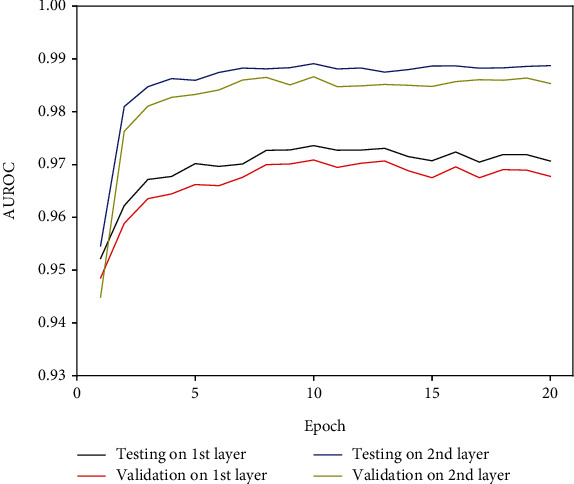
The performance of the iPTT(2 L)-CNN during crossvalidation and testing as training progressed for identifying promoters and their types as measured by AUROC.

From the high-quality benchmark data, we used 80% of them for training and 5-fold crossvalidation and the remaining 20% for testing. As shown in Figure 4, the performance of iPPT(2 L)-CNN increased as training progressed; however, when epoch was greater than 5 during 5-fold crossvalidation and testing, the area under the receiver operating characteristic curve (AUROC) values had no significant changes. We could observe that when epoch was set to 10, the first layer of iPPT(2 L)-CNN reached a maximum AUROC of 0.9709 during 5-fold crossvalidation and 0.9736 during testing. Furthermore, when epoch equaled 10, the second layer of iPPT(2 L)-CNN reached a maximum AUROC of 0.9866 during 5-fold crossvalidation and 0.9891 during testing.

**Figure 5:**
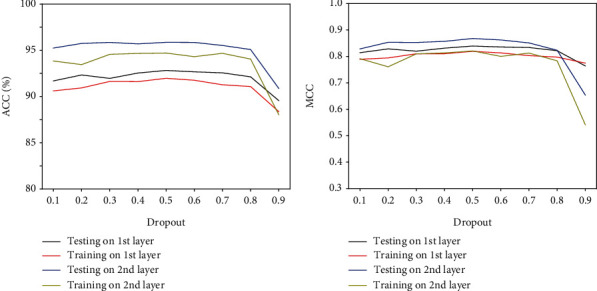
The performance of the iPTT(2 L)-CNN with different dropout probabilities.

To mitigate and avoid potential overfitting of the predictor iPPT(2 L)-CNN, we added a dropout probability for connection between two layers. As shown in Figure 5, we could observe that when dropout was set to 0.5, the iPPT(2 L)-CNN reached a maximum ACC and MCC. Adam optimizer was used to adaptively adjust for the magnitudes of the components of the gradient in our CNN architecture. We computed the loss with binary_crossentropy and saved the current model parameters so that we can select if the best model with the lowest validation loss. iPPT(2 L)-CNN was developed and tested in python 3.7, and the deep learning model CNN was implemented in Keras (v2.3.1) using the Tensorflow (v2.2.0) backend.

### 3.2. Performance of Model

**Figure 6:**
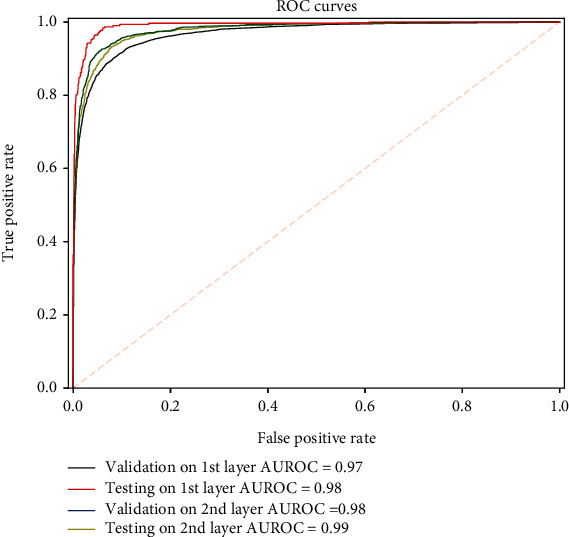
The ROC curves of the predictor iPTT (2 L)-CNN.

In the current study, 5-fold crossvalidation was used to evaluate the prediction performance of the proposed model during training. To more directly illustrate the performance of the predictor, the graph of ROC was adopted as given in Figure 6, and its AUROC value was calculated. The high AUROC value indicates that our predictor iPTT (2 L)-CNN has excellent and stable performance. The 5-fold crossvalidation results (Table 2) showed that the first layer of iPPT(2 L)-CNN achieved ACC of 91.97%, Sn of 87.26%, Sp of 94.36%, MCC of 0.8194, and AUROC of 0.97, respectively, indicating that our predictor is capable of correctly identifying whether a query sequence is of promotor or not. The second layer of iPPT(2 L)-CNN achieved ACC of 94.70%, Sn of 87.81%, Sp of 96.15%, MCC of 0.8207, and AUROC of 0.98, respectively, suggesting that our predictor can correctly identify whether a recognized promoter is of TATA or TATA-less promoter.

To evaluate the robustness and reliability of the prediction model, the prediction was also performed the aforementioned independent dataset. The iPPT(2 L)-CNN achieved a higher accuracy on these testing data as showed in Table 2. The first layer of iPPT(2 L)-CNN achieved ACC of 92.82%, Sn of 89.42%, Sp of 94.55%, MCC of 0.8394, and AUROC of 0.98, respectively. The second layer of our predictor achieved ACC of 95.86%, Sn of 94.83%, Sp of 95.86%, MCC of 0.8679, and AUROC of 0.99, respectively. The iPTT(2 L)-CNN demonstrated that the deep learning can extract complex promoter sequence characteristics and achieve significant accuracy.

### 3.3. Comparison with the Current Existing Predictor

To evaluate whether the constructed predictor is superior to the current existing methods on identifying the TATA and TATA-less promoters, we used the data of Arabidopsis and mouse to train our proposed model. These data provided by Ramzan et al. [24] were used to construct the CNNProm model. And then, 10-fold crossvalidation was employed to evaluate the prediction performance of CNNProm and iPTT(2 L)-CNN. The results listed in Table 3 showed that our proposed model iPTT(2 L)-CNN has received small increasing about Sn, Sp, and MCC, indicating that iPPT(2 L)-CNN complements CNNProm. However, in comparison to CNNProm, our proposed model can identify whether a query sequence is of promoter, instead of only directly classifying the TATA and TATA-less promoters.

### 3.4. Analysis of Predictive Capability of iPPT(2 L)-CNN on Cross-Species' Data

To further analyze the prediction performance of the proposed model iPPT(2 L)-CNN on crossspecies' data, the data of Arabidopsis and mouse provided by Ramzan et al. [24] were fed into iPPT(2 L)-CNN that was trained on the data from Zea mays. The testing results listed in Table 4 showed that the first layer of the iPPT(2 L)-CNN model tested on crossspecies' data from Arabidopsis and mouse was poor performer. The different species promoters have different sequence characteristics, and it needs to construct high-quality benchmark dataset for different species promoter. Conversely, the second layer of iPPT(2 L)-CNN had high predictive capability for crossspecies' data from Arabidopsis and mouse, illustrating significant difference between TATA and TATA-less promoters. The above results show that it is necessary to construct a two-layer predictor for first identifying the promoters and subsequently classifying the types of recognized promoters.

### 3.5. Web Server

**Figure 7:**
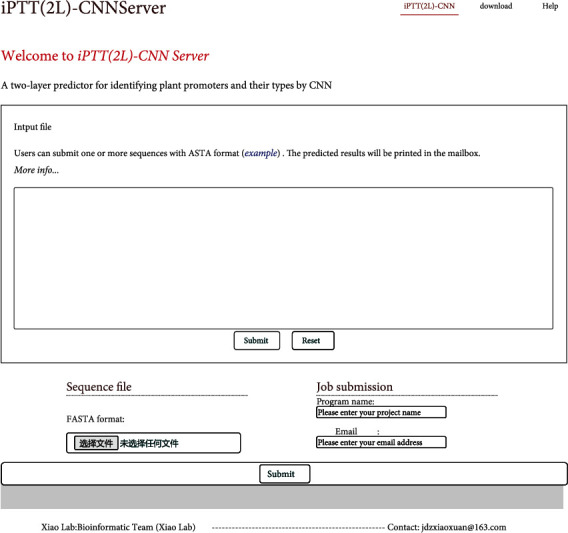
A semiscreen shot of the top page for the web server iPTT(2 L)-CNN.

User-friendly and publicly accessible web servers can not only facilitate more scholars to make relevant research but also can drive technology advances in bioinformatics and medical science. Therefore, in this study, we also established the web server for the proposed predictor, just like the web servers PEPred-Suite [43], ELM-MHC [44], and iProEP [45]. It can be accessed via the link at http://www.jci-bioinfo.cn/iPPTT(2L)-CNN.  Figure 7 shows the top page of the web server. The broad experimental scholars without computer modeling background could easily obtain the desired results only by just following the online instructions.

## 4. Conclusions

In this study, we designed a fast and effective CNN model, named iPTT(2 L)-CNN, to identify promoters and classify their types (TATA or TATA-less promoters). The robustness and good performance of the model were verified by the experiments. More importantly, we set up an online web server, which can bring great convenience to broad experimental scientists.

However, there are some limits in the proposed method. For example, we only consider the promoter sequences from Zea mays; in fact, more species should be involved. In future work, we will make efforts to collect more promoter data in plant genomes.

## Figures and Tables

**Table 1:** The specific parameters of the proposed model.

Model	Layer type	Output shape
iPTT (2 L)-CNN	Input	251 × 4
	Conv1D (300,4,1)	248 × 300
	MaxPooling1D (2,1)	247 × 300
	Conv1D (120,4,1)	244 × 120
	MaxPooling1D (2,1)	122 × 120
	Flatten	14640
	Dropout (0.5)	14640
	Dense (ReLu)	1280
	Dropout (0.5)	1280
	Dense (ReLu)	256
	Dense (sigmoid)	2

**Table 2:** The validation performance of the proposed model on training data using 5-fold crossvalidation and the testing performance of the proposed model on testing data.

Method	Layer	Sn (%)	Sp (%)	Acc (%)	Mcc
Validation	1st layer	87.26	94.36	91.97	0.8194
	2nd layer	87.81	96.15	94.70	0.8207
Testing	1st layer	89.42	94.55	92.82	0.8394
	2nd layer	94.83	95.86	95.86	0.8679

**Table 3:** Comparison of the current existing model using 10-fold crossvalidation for classifying TATA and TATA-less promoters.

Species	Type	Predictor	Sn (%)	Sp (%)	Acc (%)	Mcc
Arabidopsis	TATA	iPTT(2 L)-CNN	95.99	97.74	97.14	0.9366
		CNNProm	95.00	97.00	96.11	0.91
	TATA-less	iPTT(2 L)-CNN	94.55	96.37	95.74	0.9065
		CNNProm	94.00	94.00	93.77	0.8600
Mouse	TATA	iPTT(2 L)-CNN	95.52	97.68	97.08	0.9279
		CNNProm	97.00	97.00	97.10	0.93
	TATA-less	iPTT(2 L)-CNN	89.11	95.94	92.91	0.8513
		CNNProm	88.00	94.00	91.75	0.83

**Table 4:** The predictive performance of the constructed model iPPT(2 L)-CNN tested on cross-species' data.

Species	iPPT(2 L)-CNN	Sn (%)	Sp (%)	Acc (%)	Mcc
Arabidopsis	1st layer	44.64	84.76	72.77	0.3150
	2nd layer	92.12	92.85	92.70	0.7954
Mouse	1st layer	30.64	89.48	66.99	0.2534
	2nd layer	92.27	97.96	97.55	0.8342

## Data Availability

The benchmark dataset can be downloaded from the web http://www.jci-bioinfo.cn/iPTT(2L)-CNN/download.
